# Elementary Advice to Mothers and Nurses

**Published:** 1878-01

**Authors:** 


### Elementary Advice to Mothers and Nurses.

The duty of a mother is to preserve the life of her infant by suckling it from her own breast, or, if her health will not permit of this, by providing for it a nurse. If it be absolutely impossible to give the child human milk, or if this be insufficient in quantity it ought to be supplemented by the milk of some animal (cow goat, etc. , ) for milk is the only nourishment suitable during the early months of life. Animal milk ought to be given under those conditions which render it most like the mother's milk. It should be taken as far as possible from the same animal. It should be given, still warm, soon after it is drawn, unless it be taken fresh, in a glass which has been thoroughly cleansed between the time of milking and that of the meal. It should be boiled. It should be diluted with slightly sweetened water, warm enough to bring the mixture to the temperature of the body (37 degrees centigrade ; 98.4 Fahr. ) The dilution should be made at the time of each meal; with one-half water during the first week; one-third water during the three following weeks; one-quarter water afterwards up to the fourth month. Dating from this time it should be given warmed in a water bath, not diluted, but with the addition of a very small quantity of sugar. The hours of feeding ought to be regulated. During the day a meal every two hours is necessary, but an interval of four or five hours between the two meals from the middle of the night should be reserved for rest for the nurse. After the sixth month various milk gruels may be given or light paps of cheese farina. About the end of the first year fat (meat) soups may be taken occasionally whilst still continuing the milk. The child will thus by degrees be prepared for weaning.

Weaning.—The weaning ought only to be made after the eruption of from 12 to 16 teeth, taking into account besides the season of the year and the health of the child. Even after weaning, animal milk ought still to enter largely into the diet up to the age of two years at least.

Toilet.—Each morning, before the first meal, the child should be washed from head to foot, with water rather fresh than hot, and have his linen changed. Where needful, a hair brush and oil should be used every day to prevent the formation of "bouzet," which is only an injurious crust (dandruff). Washing of the lower part of the body should be repeated as often as it becomes soiled with urine or stools.

Clothing.—The clothing will vary so as to protect the child from the variations of the temperature. The garments should always be large enough to permit of the greatest freedom of movements. The belly-band(binder)should form part of the clothing during the first months.

Bed.—The mother and child should never sleep in the same bed. The cradle should be scrupulously clean; the air and the light should circulate freely around it.

Exercise.—During the first days the newly-born babe should be held in the arms or on the knees for some hours; but, unless in an exceptionally mild temperature, should not be taken out before the fifteenth day. After the first going out it should be carried out every day during the mildest hours. These walks, short at first, should be gradually increased, the prolonged action of pure air favoring the developement and health of the child. The day should then be divided between long sleeps and long walks at regular hours. In the intervals the child should be laid upon the floor upon a blanket, free to move and roll about. He will thus learn to raise himself alone, and to walk when the time comes, without running the risks which the use of carriages and wheeled panniers, etc., entails. The midday sleep should be continued up to the age of three years at least.—*Union Medicale du Nord-est.—Sothern Illinois Medical Association Journal.*

